# Deep Eutectic Solvents as Efficient Media for the Extraction and Recovery of Cynaropicrin from *Cynara cardunculus* L. Leaves

**DOI:** 10.3390/ijms18112276

**Published:** 2017-10-30

**Authors:** Emanuelle L. P. de Faria, Rafael S. do Carmo, Ana Filipa M. Cláudio, Carmen S. R. Freire, Mara G. Freire, Armando J. D. Silvestre

**Affiliations:** CICECO—Aveiro Institute of Materials, Chemistry Department, University of Aveiro, 3810-193 Aveiro, Portugal; emanuelle.pache@gmail.com (E.L.P.d.F.); rafaelcarmo@ua.pt (R.S.d.C.); anafmclaudio@ua.pt (A.F.M.C.); cfreire@ua.pt (C.S.R.F.); maragfreire@ua.pt (M.G.F.)

**Keywords:** deep eutectic solvent, aqueous solution, cynaropicrin, extraction, recovery

## Abstract

In recent years a high demand for natural ingredients with nutraceutical properties has been witnessed, for which the development of more environmentally-friendly and cost-efficient extraction solvents and methods play a primary role. In this perspective, in this work, the application of deep eutectic solvents (DES), composed of quaternary ammonium salts and organic acids, as alternative solvents for the extraction of cynaropicrin from *Cynara cardunculus* L. leaves was studied. After selecting the most promising DES, their aqueous solutions were investigated, allowing to obtain a maximum cynaropicrin extraction yield of 6.20 wt %, using 70 wt % of water. The sustainability of the extraction process was further optimized by carrying out several extraction cycles, reusing either the biomass or the aqueous solutions of DES. A maximum cynaropicrin extraction yield of 7.76 wt % by reusing the solvent, and of 8.96 wt % by reusing the biomass, have been obtained. Taking advantage of the cynaropicrin solubility limit in aqueous solutions, water was added as an anti-solvent, allowing to recover 73.6 wt % of the extracted cynaropicrin. This work demonstrates the potential of aqueous solutions of DES for the extraction of value-added compounds from biomass and the possible recovery of both the target compounds and solvents.

## 1. Introduction

A growing awareness of human activities on the environment, as well as the need to obtain products non-contaminated with hazardous solvents, stimulated the development of “greener extraction” processes [1]. Green technologies seek for new solvents to replace common organic ones that present inherent toxicity and volatility problems [2,3]. Several types of alternative solvents, including deep eutectic solvents (DES), have been suggested as candidates within the development of greener processes [4]. First reported by Abbot et al. [5], DES are composed of at least a hydrogen bond acceptor (HBA) and a hydrogen bond donor (HBD) species, which upon mixing establish strong hydrogen bond interactions leading to the formation of eutectic mixtures, often becoming liquid at conditions close to room temperature. The most common DES are based on cholinium chloride ([Ch]Cl) as the HBA, and HBDs comprising urea, polyols and carboxylic acids [2]. With respect to environmental and economic benefits, DES offer many advantages, including low volatility, simple preparation, low cost, and low toxicity profile [1]. As far as applications are concerned, it has been demonstrated that DES can be successfully used for the extraction of different types of natural bioactive compounds from biomass, including flavonoids [6], ginsenosides [7], anthocyanins [8], catechins [9], among others. Furthermore, it was recently demonstrated that they can also be used in the dissolution of more complex biomacromolecules, such as lignin [10]. Also worth noting, and similarly to ionic liquids [11,12,13], it was demonstrated that aqueous solutions of DES can perform better than the pure solvents [14], with inherent economic and process benefits. Finally, if benign or natural-based DES (the so called NADES) are used, these may be kept in the final formulations, in some cases also contributing to the improvement of the extracts biological properties [15,16], again with a close similarity to ILs [12].

**Figure 1:**
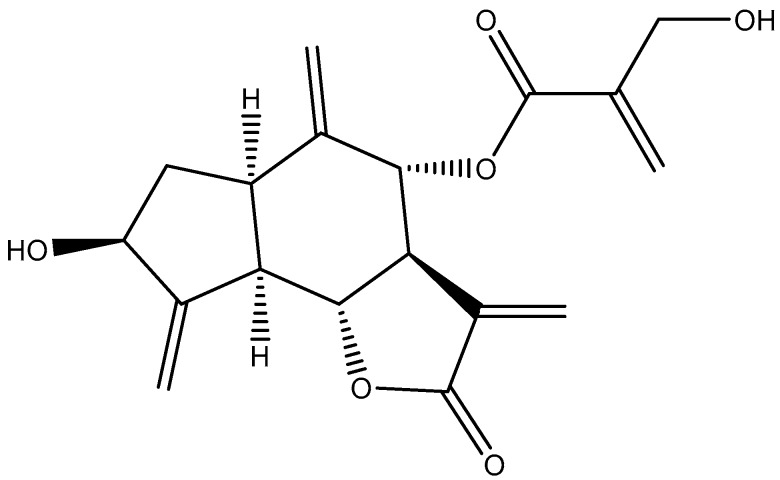
Chemical structures of cynaropicrin.

Cardoon or wild artichoke (*C. cardunculus* L., Compositae) is a perennial plant, sharing a recent common ancestor with the modern cultivated ‘‘globe artichoke”, *C. scolymus* L. [17]. Both plants have their origin in edible *Cynara* cultivars, used by early farmers in the Mediterranean basin and Macaronesia (Madeira and Canary Islands) [18]. Several bioactive compounds were already identified in *C. cardunculus* leaves and seeds [17,18,19], namely saponins, flavones, sterols, coumarins and lignans, as well as sesquiterpene lactones, such as cynaropicrin [20,21]. Cynaropicrin (Figure 1), is the main responsible for the bitter taste of *C. cardunculus* leaves, displaying a wide range of biological activities. These include anti-inflammatory [22,23], antispasmodic [24], antitrypanosomal [25], and proapoptotic [26] properties, being therefore currently used as part of crude extracts in several nutraceutical formulations.

The extraction of cynaropicrin is most often performed using conventional and in some cases toxic organic solvents, such as chloroform [27] and dichloromethane [23]. Other cynaropicrin extraction methods/solvents include, for example, water at elevated temperatures [28], or supercritical CO_2_ [29]. These methods require however the use of sophisticated equipment and are often highly energy consuming, in addition to the problems associated to the organic solvents commonly employed, which may lead to several human risks, safety issues and environmental impact [30]. To overcome some of these concerns, either regarding their environmental footprint or when used for the extraction of target compounds envisioned for human use, DES have emerged as promising alternative solvents [2].

Taking into account the biological potential of cynaropicrin, together with its abundance in *C. cardunculus* leaves (accounting for up to 87 g/kg) [23], its high commercial value [31] and nutraceutical applications, we propose here the use of DES as alternative solvents for the extraction of cynaropicrin from *C. cardunculus* leaves. An initial screening on DES (chemical structure and HBD:HBA molar ratio) was performed by the mixture of several carboxylic acids as HBD and quaternary ammonium salts as HBA. Some well-studied organic solvents were also investigated for comparison purposes. After identifying the most promising DES, the process variables (namely, solid–liquid ratio (S/L ratio), temperature (*T*), time of extraction (*t*) and percentage of water added to the DES) were optimized to maximize the cynaropicrin extraction yield. Finally, both the solvent and biomass reuse were addressed, and water was added as an anti-solvent allowing to recover the extracted cynaropicrin and to recycle the DES aqueous solution.

## 2. Results and Discussion

### 2.1. Screening of DES as Solvents for the Extraction of Cynaropicrin

**Figure 2:**
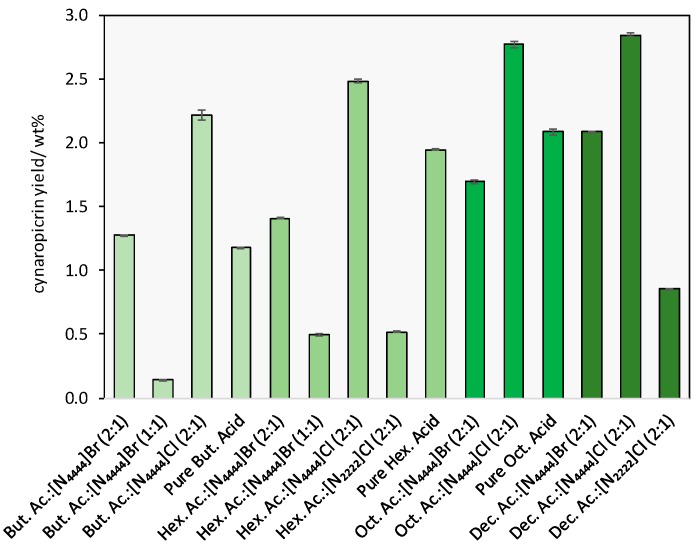
Cynaropicrin extraction yield from *C. cardunculus* leaves using several DES at different molar ratios (HBD:HBA) and the corresponding pure acids; *T* = 25 °C, *t* = 120 min and S/L ratio = 1.10. The results presented correspond to the average of three independent experiments.

Several DES were investigated to identify the best solvents for cynaropicrin extraction from *C. cardunculus* leaves. The studied DES, described in detail in the Supplementary Data (Table S1), were prepared combining HBDs (carboxylic acids) with HBAs (quaternary ammonium salts) at different molar ratios. Those identified as liquid at 25 °C were then used in the preliminary extraction assays. The respective pure acids that are liquid at the same temperature were also investigated for comparison purposes. Fixed operational conditions were used in this screening, namely a S/L ratio of 1:10, an extraction time of 120 min and a temperature of 25 °C. The results obtained are depicted in Figure 2 (detailed data is provided in the Supplementary Data—Table S2).

Cynaropicrin extraction yields obtained with DES ranged from 0.14 to 2.84 wt %. In general, there is an increase in the extraction efficiency of cynaropicrin with the increase of the carboxylic acid alkyl chain. If the HBA is kept constant, e.g., [N_4444_]Cl or [N_4444_]Br, the DES comprising butanoic and decanoic acids are the ones which exhibit lower and higher extraction yields, respectively (Figure 2). This finding is in agreement with previous observations showing that the extraction of volatile aliphatic components (namely aliphatic acids) increases with the alkyl chain length of both the HBA and HBD [32]. Furthermore, chloride-based salts lead to higher extraction yields of cynaropicrin when compared to the bromide counterparts.

Figure 2 also shows an increase in the cynaropicrin extraction yield by decreasing the amount of the HBA species, with DES based on a 2:1 molar ratio of HBD:HBA leading to a higher extraction yield, which may be due to a decrease in the viscosity of the solvent, as reported in previous studies [31,32].

The potential of the DES was further evaluated by comparison with the corresponding pure HBDs components. To this end, the cynaropicrin extraction yields using the liquid carboxylic acids at 25 °C, namely butanoic, hexanoic and octanoic acids were investigated (pure decanoic acid was not tested because it is solid at 25 °C). The results obtained show that the cynaropicrin extraction yields are higher when using DES instead of the corresponding pure HBDs. In summary, the overall results demonstrate that among the tested DES, the most efficient for the extraction of cynaropicrin from *C. cardunculus* leaves is decanoic acid:[N_4444_]Cl, in a 2:1 molar ratio.

### 2.2. Optimization of the Cynaropicrin Extraction Conditions

**Figure 3:**
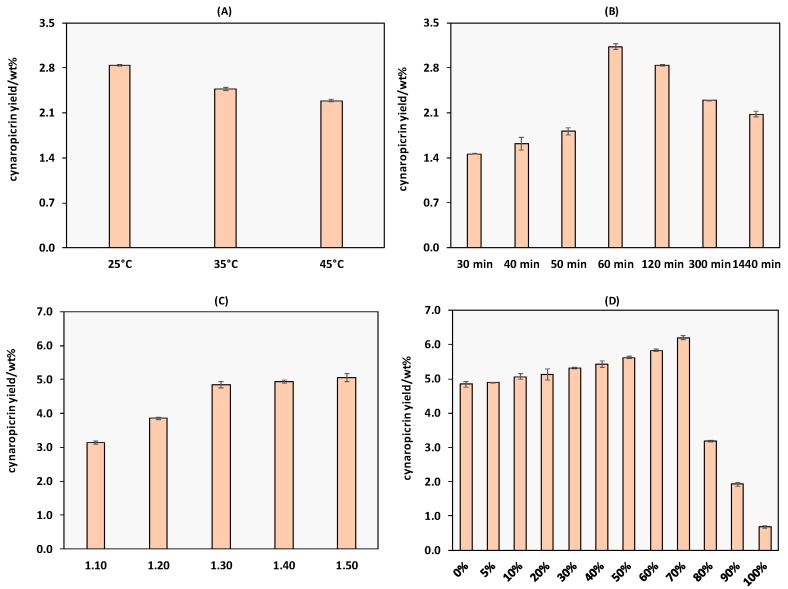
Optimization of cynaropicrin the extraction yields from *C. cardunculus* leaves using decanoic acid:[N_4444_]Cl at 2:1: (**A**) temperature (*T*); (**B**) extraction time (*t*); (**C**) solid-liquid ratio (S/L ratio); and (**D**) wt % of water in DES. The results presented correspond to the average of three independent experiments.

Extraction conditions were optimized to improve cynaropicrin extraction yield using the decanoic acid:[N_4444_]Cl 2:1 DES. The parameters studied were the extraction time (*t*, min), solid-liquid ratio (S/L ratio), temperature (*T*, °C) and water content (wt %). The influence of each variable in the cynaropicrin extraction yield is illustrated in Figure 3. The respective detailed data are provided in Supplementary Data—Tables S3–S6.

The extraction temperature was optimized, performing extractions at 25, 35 and 45 °C, while keeping constant the remaining conditions (S/L ratio of 1:10, 120 min) (Figure 3A). The maximum extraction yield, 2.84 wt %, was achieved at 25 °C (for detailed date see Supplementary Material Table S3). Higher temperatures lead to a decrease in the cynaropicrin extraction yield. Although this behaviour was expected, since an increase in temperature should promote an increased solubility and thus extraction efficiency, the lower extraction yields at higher temperatures can be due to the simultaneous dissolution of polysaccharides, which at higher temperatures lead to extremely viscous solutions and hampers the filtration process and the recovery of cynaropicrin. However, these results can be considered as promising, since high extraction yields are obtained at low temperatures with minimum energy consumptions.

The influence of the extraction time (30, 40, 50, 60, 120, 300 and 1440 min) was further evaluated, keeping constant the remaining extraction conditions (S/L ratio of 1:10 and a temperature of 25 °C (detailed data are provided in the Supplementary Data—Table S4). Considering the results presented in Figure 3B, a remarkable increase in the extraction yield up to 60 min, with a maximum extraction yield of 3.13 wt % was observed. From 60 min onwards, the extraction efficiency tends to gradually decrease. This tendency to pass through a maximum in the extraction yield has been reported before by several authors, either in the extraction of bioactive compounds from natural sources using deep eutectic solvents [33,34], or even with conventional solvents such as ethanol [35]. In the present study, these results are again most probably due to the co-dissolution of polysaccharides from biomass, since an increase in the solvent viscosity is observed after 60 min, which again is expected to hinder the recovery of cynaropicrin.

The third tested operational condition in the extraction process was the S/L ratio, an essential parameter since a lower ratio is associated with higher costs of the process and eventually solvent wastes [36]. We tested the 1:10, 1:20, 1:30, 1:40 and 1:50 S/L ratios, keeping constant the remaining conditions, namely a temperature of 25 °C and an extraction time of 60 min (detailed data are provided in the Supplementary Data—Table S5). According to the results shown in Figure 3C, the S/L ratio, shows to be a significant parameter. Decreasing the S/L ratio leads to an increase in the cynaropicrin extraction yield, reaching a maximum of 5.06 wt % using a ratio of 1:50, although between the S/L ratios of 1:30 and 1:50, the obtained yields are similar (4.84 and 5.05 wt %, respectively). A similar behaviour was observed by Wei et al., [36] who studied the extraction of bioactive compounds from *Cajanus cajan* using DES, and found that the extraction yields increase by decreasing the S/L ratio (from 1:10 to 1:40). Therefore, we propose the S/L ratio of 1:30 as the most adequate for cynaropicrin extraction since it implies a lower amount of solvent, and thus lower costs and generated wastes.

Finally, the effect of the water percentage added to the DES was investigated (Figure 3D), keeping the temperature at 25 °C, an extraction time of 60 min and a S/L ratio of 1:30 (detailed data are provided in the Supplementary Data—Table S6). An increase in the water content from 5 wt % up to 70 wt % promotes a gradual increase in the cynaropicrin extraction yield, up to a maximum of 6.20 wt %. In fact, the addition of water to DESs has shown to be advantageous either to improve the solubility or extraction of target bioactive compounds [10,32,37]. The results obtained with aqueous solutions of DES are advantageous over pure DES, either from an environmental or economic point of view when envisaging the development of large scale processes. In summary, the overall results highlight the potential of aqueous solutions of DES to extract cynaropicrin from biomass, using mild conditions (25 °C, 60 min, S/L ratio of 1:30 and 70 wt % of water added to DES).

**Figure 4:**
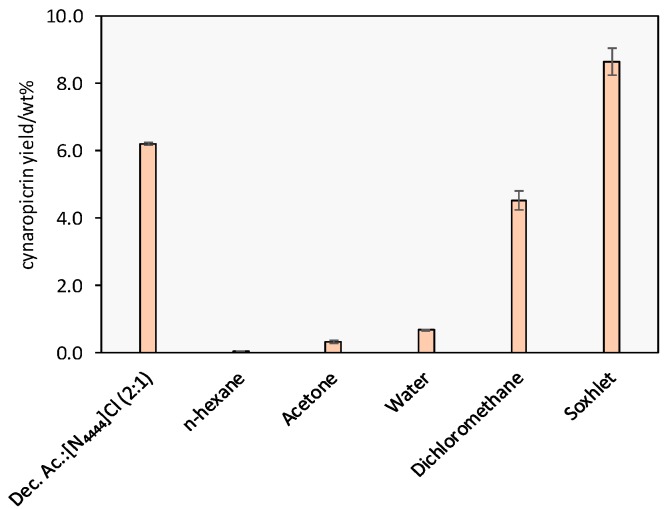
Cynaropicrin extraction yields from *C. cardunculus* leaves with the solution composed of 70% of water in DES and several molecular solvents at fixed conditions: *T* = 25 °C, *t* = 60 min, a S/L ratio = 1:30. The results presented correspond to the average of three independent experiments.

The best extraction conditions were then applied in a comparative extraction study using different organic solvents, namely *N*-hexane, acetone, and dichloromethane, commonly used in the extraction of cynaropicrin, as well as pure water. A soxhlet extraction with dichloromethane was also carried out. The results obtained are depicted in Figure 4 whereas the detailed results are provided in the Supplementary Data (Table S7). The maximum cynaropicrin extraction yield was achieved by Soxhlet extraction with dichloromethane (8.65 wt %), in agreement with previously published results [23]. The higher yields obtained with this technique, when compared to solid-liquid extractions with the same solvent (4.53 wt %) are due to the specific extraction conditions enabled by the Soxhlet extraction (high temperature and reuse of fresh solvent in multiple extraction cycles) that allows to reach the maximum extraction capacity. All the remaining solvents lead to lower cynaropicrin extraction yields when compared to those obtained with the above studied DES aqueous solution (6.20 wt %), further supporting the high potential of this type of solvents to extract cynaropicrin from *C. cardunculus* leaves under mild conditions.

### 2.3. Recyclability of the Solvent and Biomass

**Figure 5:**
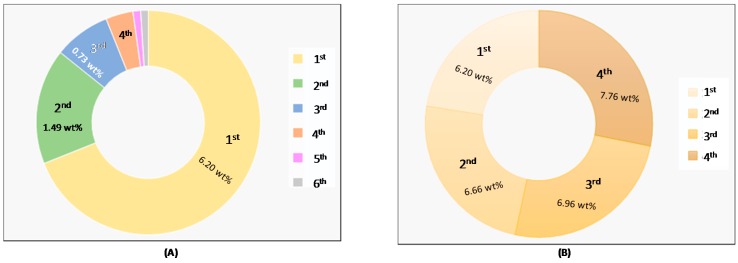
Cynaropicrin extraction yields from *C. cardunculus* leaves with the biomass (**A**) and solvent (**B**) recycle at fixed conditions: 70 wt % of water in decanoic acid:[N_4444_]Cl (2:1), *T* = 25 °C, *t* = 60 min and S/L ratio = 1:30.

Aiming to maximise the cynaropicrin recovery, the same sample of *C. cardunculus* leaves was sequentially extracted with fresh DES aqueous solutions (70 wt % of water in decanoic acid:[N_4444_]Cl 2:1) in six successive cycles, keeping the remaining optimized extraction conditions reported above. The results obtained are shown in Figure 5 and given in detail in the Supplementary Data—Table S8.

According to Figure 5, it is observed that the cynaropicrin present in the biomass sample is not fully extracted in the first cycle (6.20 wt %) and it is possible to achieve a maximum yield of 8.96 wt % after 6 extraction cycles with fresh solvent, leading to an extraction yield similar to the one obtained by Soxhlet extraction with dichloromethane (8.65 wt %). However, it should be remarked that aqueous solutions of more environmentally friendly compounds are being used in the first case, together with significantly lower temperatures and times of extraction. Overall, after the third cycle, the amount of cynaropicrin extracted is not significant to justify additional extraction cycles (taking into account the economic impact).

Similarly, the use of the same DES aqueous solution in the cynaropicrin extraction using new *C. cardunculus* leaves samples was performed, for 4 cycles. The results obtained show that the extraction yield increases in each cycle (for detailed date see Supplementary Material Table S9), however, this increase is not significant, meaning that the solvent almost reached its saturation in the first cycle.

### 2.4. Recovery of Cynaropicrin from the DES-Based Solvent

Taking into account the low solubility of cynaropicrin in water (1.75 g/L at 20 °C) [38], the recovery of cynaropicrin from the DES aqueous solution was finally evaluated using water as anti-solvent. At the end of the 3rd extraction cycle of the previous experiments (reuse/saturation of the solvent), the saturated aqueous solution of DES (0.5 mL) was then diluted with increasing volumes of water (5, 10, 15, 25 and 50 mL). The gathered data are provided in the Supplementary Data—Table S10. HPLC analysis of the remaining aqueous solution showed that up to 73.6% of the extracted cynaropicrin can be recovered by the addition of the largest volume of water. The recovery of cynaropicrin by this process further enables the recovery of the DES extraction solvent through the evaporation of the excess of water, allowing it further use.

## 3. Materials and Methods

### 3.1. Materials

*C. cardunculus* L. leaves were collected during the flowering stage, in June 2015 in an experimental field (37°59′14.25″ N, 7°55′59.64″ W) and supplied in the dry form with a granulometry between 40–60 mesh. A cynaropicrin standard was commercially purchased from Extrasynthese (Lyon, France), with a purity ≥97.5 wt %. The compounds used in the formation of the DES, composed of quaternary ammonium salts as HBA and carboxylic acids as HBD, are the following: decanoic acid (purity of 99% purity) and choline chloride ([Ch]Cl, purity of 99%), both from Acros Organics (Geel, Belgium), hexanoic acid (purity of 99%) and myristic acid (purity of 98%), both from Fluka (Sintra, Portugal), 12-hydroxystearic acid (purity of 95%) supplied by Alfa Aesar (Karlsruhe, Germany), and tetraethylammonium chloride ([N_2222_]Cl), tetrabutylammonium chloride ([N_4444_]Cl), tetrapropylammonium chloride ([N_3333_]Cl), tetrapropylammonium bromide ([N_3333_]Br), and tetrabutylammonium bromide ([N_4444_]Br), all with a purity of 98% and purchased from Sigma-Aldrich (Sintra, Portugal). Common organic solvents were also tested as extraction media, namely acetone and dichloromethane with a purity of ≥99.99%, and *n*-hexane with a purity of 95%, all acquired from Sigma-Aldrich (Sintra, Portugal).

### 3.2. DES Preparation

The preparation of DES was carried out by adding a known mass of HBD and HBA to a closed glass vial, where the solid mixture was homogenized by heating under constant stirring. The sample was heated gradually until the mixture formed a clear liquid inside the vial, and was left for 1 h at this temperature under constant stirring. DES samples were then placed under constant stirring under vacuum at a temperature of 25 °C for a minimum period of 48 h to remove possible volatile impurities. The list containing all DES prepared and used is provided in the Supplementary Data. Aqueous solutions of DES were prepared gravimetrically, ± 10^−4^ g.

### 3.3. Cynaropicrin Extraction

Solid-liquid extractions of cynaropicrin from *C. cardunculus* leaves were carried out using a home-made aluminum dry oven, with controlled stirring and able to keep the temperature within ± 0.5 °C. In all experiments stirring was kept at 1000 rpm (120 g-force considering the rotor Radius of the 10.70 cm) All the reported essays were performed in triplicate with less than 5% variability. The extraction conditions ascertained and optimized to increase the cynaropicrin extraction yield were: temperature (25, 35 and 45 °C); extraction time (30, 40, 50, 60, 120, 300 and 1440 min); solid-liquid (S/L) ratio (weight of biomass per weight of solvent, 1:10, 1:20, 1:30, 1:40 and 1:50); and percentage weight fraction of water added to the DES (0, 5, 10, 20, 30, 40, 50, 60, 70 and 100 wt %). For comparison purposes, the optimized conditions were applied using the conventional organic solvents and water. A sample of *C. cardunculus* leaves (5 g dry weight) was also Soxhlet extracted with dichloromethane (150 mL) for 7 h for comparison. After the extraction step, samples were centrifuged (Centrifuge 5804, Eppendorf), and the supernatant containing cynaropicrin filtered using a 0.20 μm syringe filter acquired at GE healthcare, Whatman. A 200 µL aliquot was taken from the supernatant of each assay, diluted with 800 µL of methanol, and filtered again. The quantification of cynaropicrin in each solution was carried out by HPLC-DAD (Shimadzu, model PROMINENCE). HPLC analyses were performed with an analytical C18 reversed-phase column (250 × 4.60 mm), kinetex 5 μm C18 100 A, from Phenomenex. The mobile phase consisted of 75% (*v*/*v*) of water and 25% (*v*/*v*) of acetonitrile. The separation was conducted in isocratic mode, at a flow rate of 0.5 mL·min^−1^, and using an injection volume of 10 μL. DAD was set at 198 nm. The column oven and the autosampler were operated at a controlled temperature of 30 °C. The quantification of cynaropicrin was performed based on a calibration curve prepared using the pure cynaropicrin sample (sample) dissolved in methanol, *R*^2^ = 0.9993. The cynaropicrin extraction yield is expressed as the percentage ratio between the weight of cynaropicrin and the total weight of the dried biomass. The results presented along the manuscript correspond to the average of three independent experiments with an associated error of <5% in the extraction yield.

### 3.4. Recyclability of the Solvent and Biomass

In order to develop a more sustainable extraction-recovery process, both 4 successive cycles of extraction using the same solvent and 6 new aqueous solutions of DES were applied to the same biomass. The main objective of this study was to appraise the solvent saturation and to infer the maximum amount of the target compound present in the biomass.

### 3.5. Recovery of the Extracted Cynaropicrin

After the 4 extraction cycles with the reuse of the same aqueous solution of DES, which allowed the solvent saturation, water was added as an anti-solvent, inducing the precipitation of cynaropicrin. Different amounts of added water were investigated; 0.5 mL of the DES aqueous solutions containing cynaropicrin were diluted in 5, 10, 15, 25 and 50 mL of distilled water. After the addition of water, the solution was centrifuged at 6000 rpm (4307 g-force considering the rotor Radius of the 10.70 cm) for 30 min, and vacuum filtered with a 0.45 μm microporous membrane. The remaining cynaropicrin in the solvent was quantified by HPLC-DAD as described before. Further, the solid residue was dried in an air oven at 50 °C for 2 days. A sample of the recovered precipitate was redissolved in 9 mL of methanol and cynaropicrin identified and quantified by HPLC-DAD.

## 4. Conclusions

The use of DES and their aqueous solutions have been investigated as alternative solvents for the extraction of the high-value cynaropicrin from *C. cardunculus* leaves. The use of long chain aliphatic organic acids as HBD, and chloride quaternary ammonium salts as HBA, allows to achieve improved cynaropicrin extraction yields, where the combination of decanoic acid:[N_4444_]Cl 2:1 was found to be the most efficient. Several extraction conditions were then optimized, namely the extraction time, S/L ratio, temperature and content of water, leading to a maximum extraction yield of 6.20 wt % of cynaropicrin using the decanoic acid:[N_4444_]Cl (2:1) aqueous solution (70 wt % of water), at 25 °C, 60 min, and a S/L ratio of 1:30. Aiming the development of more sustainable extraction processes the reuse of the solvent and of the biomass were also evaluated. Under the optimized conditions and with three cycles of fresh DES-water solvent, it was obtained an extraction yield of cynaropicrin of 8.96 wt %, a similar value to that obtained with volatile organic solvents under soxhlet extractions, yet applying milder conditions of temperature and time. Finally, 73.6% of the extracted cynaropicrin was recovered by the addition of water to the aqueous solutions of DES. In summary, DES and their aqueous solutions are remarkable alternatives to volatile organic solvents for the extraction of cynaropicrin from the *C. cardunculus* leaves, and are therefore envisaged as promising candidates for the extraction of other compounds with nutraceutical interest.

## Supplementary Materials

Supplementary materials can be found at www.mdpi.com/1422-0067/18/11/2276/s1.

## References

[1] Radošević K., Ćurko N., Srček V.G., Bubalo M.C., Tomašević M., Ganić K.K., Radojčić Redovniković I. (2016). Natural deep eutectic solvents as beneficial extractants for enhancement of plant extracts bioactivity. Food Sci. Technol..

[2] Paiva A., Craveiro R., Aroso I., Martins M., Reis R.L., Duarte A.R.C. (2014). Natural deep eutectic solvents—Solvents for the 21st century. ACS Sustain. Chem. Eng..

[3] Jessop P.G. (2011). Searching for green solvents. Green Chem..

[4] Bubalo M.C., Ćurko N., Tomašević M., Ganić K.K., Redovnikovic I.R. (2016). Green extraction of grape skin phenolics by using deep eutectic solvents. Food Chem..

[5] Abbott A.P., Boothby D., Capper G., Davies D.L., Rasheed R.K. (2004). Deep Eutectic Solvents Formed between Choline Chloride and Carboxylic Acids:  Versatile Alternatives to Ionic Liquids. JACS Artic..

[6] Nam M.W., Zhao J., Lee M.S., Jeong J.H., Lee J. (2015). Enhanced extraction of bioactive natural products using tailor-made deep eutectic solvents: Application to flavonoid extraction from *Flos sophorae*. Green Chem..

[7] Jeong K.M., Lee M.S., Nam M.W., Zhao J., Jin Y., Lee D.K., Kwon S.W., Jeong J.H., Lee J. (2015). Tailoring and recycling of deep eutectic solvents as sustainable and efficient extraction media. J. Chromatogr. A.

[8] Jeong K.M., Zhao J., Jin Y., Heo S.R., Han S.Y., Yoo D.E., Lee J. (2015). Highly efficient extraction of anthocyanins from grape skin using deep eutectic solvents as green and tunable media. Arch. Pharm. Res..

[9] Li J., Han Z., Yu B. (2015). Efficient extraction of major catechins in *Camellia sinensis* leaves using green choline chloride-based deep eutectic solvents. RSC Adv..

[10] Soares B., Tavares D.J.P., Amaral J.L., Silvestre A.J.D., Freire C.S.R., Coutinho J.A.P. (2017). Enhanced solubility of lignin monomeric model compounds and technical lignins in aqueous solutions of deep eutectic solvents. ACS Sustain. Chem. Eng..

[11] Cláudio A.F.M., Ferreira A.M., Freire M.G., Coutinho J.A.P. (2013). Enhanced extraction of caffeine from guarana seeds using aqueous solutions of ionic liquids. Green Chem..

[12] Ferreira A.M., Morais E.S., Leite A.C., Mohamadou A., Holmbom B., Holmbom T., Neves B.M., Coutinho J.A.P., Freire M.G., Silvestre A.J.D. (2017). Enhanced Extraction and Biological Activity of 7-hydroxymatairesinol obtained from Norway Spruce knots using Aqueous Solutions of Ionic Liquids. Green Chem..

[13] Faria E.L.P., Shabudin S.V., Cláudio A.F.M., Valega M., Domingues F.M.J., Freire C.S.R., Silvestre A.J.D., Freire M.G. (2017). Aqueous solutions of surface-active ionic liquids: Remarkable alternative solvents to improve the solubility of triterpenic acids and their extraction from biomass. ACS Sustain. Chem. Eng..

[14] Dai Y., Witkamp G.J., Verpoorte R., Choi Y.H. (2013). Natural deep eutectic solvents as a new extraction media for phenolic metabolites in *Carthamus tinctorius* L.. Anal. Chem..

[15] Dai Y., Van Spronsen J., Witkamp G.J., Verpoorte R., Choi Y.H. (2013). Natural deep eutectic solvents as new potential media for green technology. Anal. Chim. Acta.

[16] Choi Y.H., Van Spronsen J., Dai Y., Verberne M., Hollmann F., Arends I.W.C.E., Witkamp G.-J., Verpoorte R. (2011). Are Natural Deep Eutectic Solvents the Missing Link in Understanding Cellular Metabolism and Physiology?. Plant Physiol..

[17] Wiklund A. (1992). The genus *Cynara* L. (Asteraceae-Cardueae). Bot. J. Linn. Soc..

[18] Lerna A., Mauromicale G. (2010). *Cynara cardunculus* L. genotypes as a crop for energy purposes in a Mediterranean environment. Biomass Bioenergy.

[19] Koubaa I., Damak M. (2003). A new dilignan from *Cynara cardunculus*. Fitoterapia.

[20] Pinelli P., Agostini F., Comino C., Lanteri S., Portis E., Romani A. (2007). Simultaneous quantification of caffeoyl esters and flavonoids in wild and cultivated cardoon leaves. Food Chem..

[21] Sevcíková P., Glatz Z., Slanina J. (2002). Analysis of artichoke (*Cynara cardunculus* L.) extract by means of micellar electrokinetic capillary chromatography. Electrophoresis.

[22] Elsebai M.F., Mocan A., Atanasov A. (2016). Cynaropicrin: A comprehensive research review and therapeutic potential as an anti-hepatitis C virus agent. Front. Pharmacol..

[23] Ramos P.A.B., Guerra A.R., Guerreiro O., Freire C.S.R., Silva A.M.S., Duarte M.F., Silvestre A.J.D. (2013). Lipophilic extracts of *Cynara cardunculus* L. var. *altilis* (DC): A source of valuable bioactive terpenic compounds. J. Agric. Food Chem..

[24] Emendörfer F., Emendörfer F., Bellato F., Noldin V.F., Cechinel-Filho V., Yunes R.A., Delle Monache F., Cardozo A.M. (2005). Antispasmodic activity of fractions and cynaropicrin from *Cynara scolymus* on guinea-pig ileum. Biol. Pharm. Bull..

[25] Zimmermann S., Adams M., Julianti T., Hata-Uribe Y., Brun R., Hamburger M. (2010). HPLC-based activity profiling for new antiparasitic leads: In vitro and in vivo antitrypanosomal activity of cynaropicrin. Plant. Med..

[26] Cho J.Y., Kim A.R., Jung J.H., Chun T., Rhee M.H., Yoo E.S. (2004). Cytotoxic and pro-apoptotic activities of cynaropicrin, a sesquiterpene lactone, on the viability of leukocyte cancer cell lines. Eur. J. Pharmacol..

[27] Bhattacharyya P.R., Barua N.C., Ghosh A.C. (1995). Cynaropicrin from *Tricholepis glaberrima*: A potential insect feeding deterrent compound. Ind. Crops Prod..

[28] Schneider G., Thiele K. (1974). Distribution of the bitter principle cynaropicrin in *Cynara*. Plant Biochem..

[29] Sovová H., Opletal L., Sajfrtová M., Bártlová M. (2008). Supercritical fluid extraction of cynaropicrin and 20-hydroxyecdysone from *Leuzea carthamoides* DC. J. Sep. Sci..

[30] Clark J.H., Luque R., Matharu A.S. (2012). Green chemistry, biofuels, and biorefinery. Annu. Rev. Chem. Biomol. Eng..

[31] Sigma-Aldrich. https://www.sigmaaldrich.com.

[32] Van Osch D.J.G.P., Zubeir L.F., Van Den Bruinhorst A., Rocha M.A.A., Kroon M.C. (2015). Hydrophobic deep eutectic solvents as water-immiscible extractants. Green Chem..

[33] Tang B., Park H.E., Row K.H. (2015). Simultaneous extraction of flavonoids from *Chamaecyparis obtusa* using deep eutectic solvents as additives of conventional extractions solvents. J. Chromatogr. Sci..

[34] Qi X.L., Peng X., Huang Y.Y., Li L., Wei Z.F., Zu Y.G., Fu Y.J. (2015). Green and efficient extraction of bioactive flavonoids from *Equisetum palustre* L. by deep eutectic solvents-based negative pressure cavitation method combined with macroporous resin enrichment. Ind. Crops. Prod..

[35] Trendafilova A., Chanev C., Todorova M. (2010). Ultrasound-assisted extraction of alantolactone and isoalantolactone from *Inula helenium* roots. Pharmacogn. Mag..

[36] Wei Z., Qi X., Li T., Luo M., Wang W., Zu Y., Fu Y. (2015). Application of natural deep eutectic solvents for extraction and determination of phenolics in *Cajanus cajan* leaves by ultra performance liquid chromatography. Sep. Purif. Technol..

[37] Park H.E., Tang B., Row K.H. (2014). Application of Deep Eutectic Solvents as Additives in Ultrasonic Extraction of Two Phenolic Acids from Herba *Artemisiae scopariae*. Anal. Lett..

[38] Chemspider The Free Chemical Database. http://www.chemspider.com.

