# Supplementary material for: Deep Eutectic Solvents as Efficient Media for the Extraction and Recovery of Cynaropicrin from Cynara cardunculus L. Leaves
Source: Int J Mol Sci. 2017 Oct 30;18(11):2276. doi: 10.3390/ijms18112276 (PMC5713246; doi:10.3390/ijms18112276)
Supplement: Supplementary file 1 [file ijms-18-02276-s001.pdf]

# Supplementary data

# Deep Eutectic Solvents as Efficient Media for the Extraction and Recovery of Cynaropicrin from Cynara cardunculus L. leaves

**Emanuelle L.P. de Faria [1], Rafael S. do Carmo [1], Ana Filipa M. Cláudio [1], Carmen S.R. Freire [1], Mara G. Freire [1] and Armando J.D. Silvestre [1,*]**

[1] CICECO – Aveiro Institute of Materials, Chemistry Department, University of Aveiro, Campus de Santiago, 3810-193 Aveiro, Portugal

*Corresponding author: armsil@ua.pt; tel: + 351 234370701; Fax: +351 234370084

**Table S1.** Prepared DES, HBD:HBA ratio and physical state at room temperature (ca. 25⁰C).

| Hydrogen bond donor | Hydrogen bond Acceptor | HBD:HBA Ratio | Physical state |
|---|---|---|---|
| Decanoic acid | Choline chloride | 1:1 / 2:1 / 1:2 | Solid |
| Hexanoic acid | Choline chloride | 2:1 | Solid |
| Hexanoic acid | Choline chloride | 4:1 | Solid |
| Hexanoic acid | $[N_{2222}]Br$ | 2:1 | Solid |
| Decanoic acid | $[N_{2222}]Br$ | 2:1 | Solid |
| Hexanoic acid | $[N_{3333}]Br$ | 2:1 | Solid |
| Decanoic acid | $[N_{3333}]Br$ | 2:1 | Solid |
| Butanoic acid | $[N_{4444}]Br$ | 2:1 | Liquid |
| Butanoic acid | $[N_{4444}]Br$ | 1:1 | Liquid |
| Butanoic acid | $[N_{4444}]Br$ | 1:2 | Solid |
| Caprylic acid/ hexanoic | $[N_{4444}]Br$ | 1:1 | Liquid |
| Caprylic acid/ hexanoic | $[N_{4444}]Br$ | 2:1 | Liquid |
| Caprylic acid/ hexanoic | $[N_{4444}]Br$ | 1:2 | Solid |
| Octanoic acid | $[N_{4444}]Br$ | 2:1 | Liquid |
| Octanoic acid | $[N_{4444}]Br$ | 1:1 | Solid |
| Octanoic acid | $[N_{4444}]Br$ | 1:2 | Solid |
| Decanoic acid | $[N_{4444}]Br$ | 2:1 | Liquid |
| Decanoic acid | $[N_{4444}]Br$ | 1:1 | Solid |
| Decanoic acid | $[N_{4444}]Br$ | 1:2 | Solid |

| | | | |
|---|---|---|---|
| Lauric acid/ dodecanoic | $[N_{4444}]Br$ | 2:1 | Liquid |
| Lauric acid/ dodecanoic | $[N_{4444}]Br$ | 1:1 | Solid |
| Lauric acid/ dodecanoic | $[N_{4444}]Br$ | 1:2 | Solid |
| Miristic acid/ tetradecanoic | $[N_{4444}]Br$ | 2:1 | Solid |
| Hexanoic acid | $[N_{2222}]Cl$ | 2:1 | Liquid |
| Decanoic acid | $[N_{2222}]Cl$ | 2:1 | Liquid |
| Caprilic acid / hexanoic | $[N_{3333}]Cl$ | 2:1 | Liquid |
| Caprilic acid/ Hexanoic | $[N_{3333}]Cl$ | 1:1 | Solid |
| Caprilic acid/ Hexanoic | $[N_{3333}]Cl$ | 1:2 | Solid |
| Decanoic acid | $[N_{3333}]Cl$ | 2:1 | Liquid |
| Lauric acid/ dodecanoic | $[N_{3333}]Cl$ | 2:1 | Liquid |
| Lauric acid/ dodecanoic | $[N_{3333}]Cl$ | 1:1 | Solid |
| Lauric acid/ dodecanoic | $[N_{3333}]Cl$ | 1:2 | Solid |
| Butanoic acid | $[N_{4444}]Cl$ | 2:1 | Liquid |
| Butanoic acid | $[N_{4444}]Cl$ | 1:1 | Solid |
| Butanoic acid | $[N_{4444}]Cl$ | 1:2 | Solid |
| Hexanoic acid | $[N_{4444}]Cl$ | 2:1 | Liquid |
| Hexanoic acid | $[N_{4444}]Cl$ | 1:1 | Solid |
| Hexanoic acid | $[N_{4444}]Cl$ | 1:2 | Solid |
| Octanoic acid | $[N_{4444}]Cl$ | 2:1 | Liquid |
| Octanoic acid | $[N_{4444}]Cl$ | 1:1 | Solid |

| | | | |
|---|---|---|---|
| Octanoic acid | $[N_{4444}]Cl$ | 1:2 | Solid |
| Decanoic acid | $[N_{4444}]Cl$ | 2:1 | Liquid |
| Decanoic acid | $[N_{4444}]Cl$ | 1:1 | Liquid |
| Decanoic acid | $[N_{4444}]Cl$ | 1:2 | Solid |
| 12-hydroxystearic acid | $[N_{4444}]Br$ | 1:1 / 2:1 / 1:2 | Solid |
| 12-hydroxystearic acid | $[N_{3333}]Cl$ | 1:1 / 2:1 / 1:2 | Solid |
| 12-hydroxystearic acid | Choline chloride | 1:1 / 2:1 / 1:2 | Solid |

**Table S2.** Weight fraction percentage (extraction yield, wt.%) of cynaropicrin from the leaves of *Cynara cardunculus L.* with several DES at different molar ratio and fixed conditions, S/L ratio= 1:10, T = 25°C and t = 120 min.

| DES | Cynaropicrin (wt.%) |
|---|---|
| **But. Acid:$[N_{4444}]Br$ (2:1)** | 1.273 ± 0.003 |
| **But. Acid:$[N_{4444}]Br$ (1:1)** | 0.138 ± 0.003 |
| **But. Acid:$[N_{4444}]Cl$ (2:1)** | 2.216 ± 0.036 |
| **But. Acid:$[N_{2222}]Cl$ (2:1)** | ND |
| **Pure But. Acid** | 1.175 ± 0.000 |
| **Hex. Acid:$[N_{4444}]Br$ (2:1)** | 1.405 ± 0.004 |
| **Hex. Acid:$[N_{4444}]Br$ (1:1)** | 0.498 ± 0.010 |
| **Hex. Acid:$[N_{4444}]Cl$ (2:1)** | 2.483 ± 0.017 |
| **Hex. Acid:$[N_{2222}]Cl$ (2:1)** | 0.518 ± 0.007 |
| **Pure Hex. Acid** | 1.944 ± 0.002 |

| | |
|---|---|
| **Oct. Acid:[$N_{4444}$]Br (2:1)** | 1.694 ± 0.016 |
| **Oct. Acid:[$N_{4444}$]Br (1:1)** | ND |
| **Oct. Acid:[$N_{4444}$]Cl (2:1)** | 2.768 ± 0.023 |
| **Oct. Acid:[$N_{2222}$]Cl (2:1)** | ND |
| **Pure Oct. Acid** | 2.083 ± 0.028 |
| **Dec. Acid:[$N_{4444}$]Br (1:1)** | ND |
| **Dec. Acid:[$N_{4444}$]Br (2:1)** | 2.085 ± 0.001 |
| **Dec. Acid:[$N_{4444}$]Cl (2:1)** | 2.842 ± 0.014 |
| **Dec. Acid:[$N_{2222}$]Cl (2:1)** | 0.851 ± 0.003 |
| **Pure Dec. Acid** | ND |

*ND – Not determined.

**Table S3.** Weight fraction percentage (extraction yield, wt.%) of cynaropicrin from the leaves of *Cynara cardunculus L.* with decanoic acid:[$N_{4444}$]Cl (2:1) at different temperatures and other fixed conditions (S/L ratio = 1:10 and t = 120 min).

| **DES/Temperature** | **Cynaropicrin (wt.%)** | | |
|---|---|---|---|
| | **25 $^{0}$C** | **35 $^{0}$C** | **45 $^{0}$C** |
| **Decanoic acid:[$N_{4444}$]Cl** | 2.842 ± 0.014 | 2.473 ± 0.030 | 2.292 ± 0.022 |

**Table S4.** Weight fraction percentage (extraction yield, wt.%) of cynaropicrin from the leaves of *Cynara cardunculus L.* with decanoic acid:[$N_{4444}$]Cl (2:1) in different extraction time and fixed conditions, S/L ratio = 1:10 and T = 25°C.

| **DES/Extraction time** | **Cynaropicrin (wt.%)** | | | | | | |
|---|---|---|---|---|---|---|---|
| | **30 min** | **40 min** | **50 min** | **60 min** | **120 min** | **300 min** | **1440 min** |
| **Decanoic acid:[$N_{4444}$]Cl** | 1.464 ± 0.005 | 1.614 ± 0.099 | 1.812 ± 0.052 | 3.130 ± 0.044 | 2.842 ± 0.014 | 2.295 ± 0.000 | 2.080 ± 0.042 |

**Table S5.** Optimization of weight fraction percentage of cynaropicrin extracted from the leaves of *Cynara cardunculus L.* with decanoic acid:[$N_{4444}$]Cl (2:1) at solid-liquid ratio and other fixed conditions (T = 25°C and t = 60 min).

| **DES/Solid-liquid ratio** | **Cynaropicrin (wt.%)** | | | | |
|---|---|---|---|---|---|
| | **1:10** | **1:20** | **1:30** | **1:40** | **1:50** |
| **Decanoic acid:[$N_{4444}$]Cl** | 3.130 ± 0.044 | 3.853 ± 0.026 | 4.842 ± 0.086 | 4.942 ± 0.045 | 5.055 ± 0.117 |

**Table S6.** Weight fraction percentage (extraction yield, wt.%) of cynaropicrin from the leaves of *Cynara cardunculus L.* using aqueous solutions of decanoic acid:[$N_{4444}$]Cl (2:1), and other fixed conditions (T = 25°C, S/L ratio = 1:30 and t = 60 min).

| **DES/Added water (wt.%)** | **Cynaropicrin (wt.%)** | | | | | | | | | | | |
|---|---|---|---|---|---|---|---|---|---|---|---|---|
| | **0** | **5** | **10** | **20** | **30** | **40** | **50** | **60** | **70** | **80** | **90** | **Pure water** |
| **Decanoic acid:[$N_{4444}$]Cl** | 4.842 ± 0.086 | 4.896 ± 0.012 | 5.065 ± 0.080 | 5.134 ± 0.164 | 5.309 ± 0.021 | 5.431 ± 0.089 | 5.622 ± 0.030 | 5.832 ± 0.030 | 6.202 ± 0.048 | 3.188 ± 0.025 | 1.928 ± 0.056 | 0.676 ± 0.036 |

**Table S7.** Weight fraction percentage (extraction yield, wt.%) of cynaropicrin from the leaves of *Cynara cardunculus L.* with several solvents and decanoic acid:[$N_{4444}$]Cl (2:1) (70 wt.% of water) at the following fixed conditions: S/L ratio = 1:30, t = 60 min and T = 25°C.

| Solvent | Cynaropicrin (wt.%) |
|---|---|
| **Decanoic acid:[$N_{4444}$]Cl** | 6.202 ± 0.048 |
| **n-hexane** | 0.037 ± 0.010 |
| **Acetone** | 0.346 ± 0.045 |
| **$H_2O$** | 0.676 ± 0.017 |
| **Dichloromethane** | 4.529 ± 0.266 |
| **Soxhlet** | 8.652 ± 0.407 |

**Table S8.** Weight fraction percentage (extraction yield, wt.%) of cynaropicrin from the leaves of *Cynara cardunculus L.* with the biomass recycle at fixed conditions (S/L ratio= 1:30, t = 60 min, T = 25°C).

| Cycle | Cynaropicrin (wt.%) |
|---|---|
| **1$^{st}$** | 6.202 ± 0.048 |
| **2$^{nd}$** | 1.491 ± 0.003 |
| **3$^{rd}$** | 0.732 ± 0.018 |
| **4$^{th}$** | 0.351 ± 0.003 |
| **5$^{th}$** | 0.103 ± 0.009 |
| **6$^{th}$** | 0.097 ± 0.012 |

**Table S9.** Weight fraction percentage (extraction yield, wt.%) of cynaropicrin from the leaves of *Cynara cardunculus L.* with the aqueous solution of DES recycle at fixed conditions (S/L ratio = 1:30, t = 60 min and T = 25°C)

| Cycle | Cynaropicrin (wt.%) |
|---|---|
| **1st** | 6.202 ± 0.048 |
| **2nd** | 6.664 ± 0.009 |
| **3rd** | 6.958 ± 0.109 |
| **4th** | 7.760 ± 0.093 |

**Table S10.** Amount of water (mL) added and e percentage of precipitation/recovery of cynaropicrin (0.5 mL of the DES-water solution used).

| Amount of water (mL) | Cynaropicrin recovery (%) |
|---|---|
| **5** | 38.26 |
| **10** | 48.28 |
| **15** | 52.51 |
| **25** | 65.70 |
| **50** | 73.61 |
